# The Mediating Role of Resilience in the Relationship between Big Five Personality and Anxiety among Chinese Medical Students: A Cross-Sectional Study

**DOI:** 10.1371/journal.pone.0119916

**Published:** 2015-03-20

**Authors:** Meng Shi, Li Liu, Zi Yue Wang, Lie Wang

**Affiliations:** 1 English Department, School of Basic Medicine, China Medical University, Shenyang, People’s Republic of China; 2 Department of Social Medicine, School of Public Health, China Medical University, Shenyang, People’s Republic of China; University of Vienna, AUSTRIA

## Abstract

**Backgrounds:**

The psychological distress of medical students is a major concern of public health worldwide. However, few studies have been conducted to evaluate anxiety symptoms of medical students in China. The purpose of this study was to investigate the anxiety symptoms among Chinese medical students, to examine the relationships between big five personality traits and anxiety symptoms among medical students, and to explore the mediating role of resilience in these relationships.

**Methods:**

This multicenter cross-sectional study was conducted in June 2014. Self-reported questionnaires consisting of the Zung Self-Rating Anxiety Scale (SAS), Big Five Inventory (BFI), Wagnild and Young Resilience Scale (RS-14) and demographic section were distributed to the subjects. A stratified random cluster sampling method was used to select 2925 medical students (effective response rate: 83.57%) at four medical colleges and universities in Liaoning province, China. Asymptotic and resampling strategies were used to explore the mediating role of resilience.

**Results:**

The prevalence of anxiety symptoms was 47.3% (SAS index score≥50) among Chinese medical students. After adjusting for the demographic factors, the traits of agreeableness, conscientiousness and openness were all negatively associated with anxiety whereas neuroticism was positively associated with it. Resilience functioned as a mediator in the relationships between agreeableness/conscientiousness/openness and anxiety symptoms.

**Conclusions:**

Among Chinese medical students, the prevalence of anxiety symptoms was high and resilience mediated the relationships between big five personality traits and anxiety symptoms. Identifying at-risk individuals and undertaking appropriate intervention strategies that focus on both personality traits and resilience might be more effective to prevent and reduce anxiety symptoms.

## Introduction

The psychological and mental well-being of medical students is one of public health concerns worldwide as it is correlated with the quality of health care they will provide in the future [1]. However, this population has been shown to be particularly vulnerable to psychological distress [2]. Besides depression, the prevalence of anxiety symptoms among medical students is reported to be high in many studies [3–6]. Excessive and persistent anxiety symptoms may develop into anxiety disorders [7]. Anxiety symptoms and related disorders can lead to poor academic performance, school dropout, suicidal ideation and attempts among students [6,8,9]. In addition, as one of the most common psychiatric conditions, anxiety poses a great burden on families and society, particularly primary health care services [10,11]. In fact, the prevalence of anxiety disorders has kept rising over the past few decades and has become the seventh most burdensome condition among all diseases and injuries worldwide [12].

Anxiety has been one of the most frequently studied internalizing problems among medical students [2,13]. It is revealed that individual differences in personality traits may play a role in the development of anxiety symptoms [14]. The personality traits model of five-factor model (FFM) recognizes that personality traits are hierarchically ordered from many specific characteristics to five general traits, which consist of extraversion, neuroticism, conscientiousness, agreeableness, and openness [15]. Previous studies have demonstrated that personality traits, especially neuroticism and extraversion, are related to anxiety. Specifically, while neuroticism is shown to be a predictor of anxiety symptoms, extraversion seems to protect against the symptoms [14,16–19]. However, as-of-yet only one study has examined the correlations between big five personality traits and anxiety symptoms among medical students. In the study by Bunevicius et al. [20], the severity of anxiety symptoms among medical students was found to be negatively related to emotional stability.

Although the correlations between anxiety and personality traits have been examined among junior physicians and medical students [18,20], the mechanisms behind the correlations still remain largely unknown. Identifying the mechanisms through which different personality dimensions confer risks has significant implications for the development of interventions aimed at preventing and reducing anxiety symptoms. As positive psychology is being increasingly used to prevent and treat mental health problems, resilience, one of the state-like components of psychological capital, may play a role in the relationships between personality traits and anxiety. Although the complexity of defining resilience has been widely recognized [21], based on extensive literature reviews and concept analysis, it refers to the process of negotiating, managing and adapting to significant sources of stress and trauma [22]. Resilience was found to be negatively associated with neuroticism, and positively related to extraversion and conscientiousness in a sample of American college students. All the three personality traits contributed significantly to the prediction of resilience [23]. In addition, individual differences in personality traits were found to explain 39% of the variance in resilience among 479 Australian family practitioners [24]. Meanwhile, resilience has also been found to be negatively related to anxiety among Australian general practitioners [25] and anxiety disorders among the general public [26]. Kötter et al. [27] showed that the cultivation of resilience could be a promising approach to solving deteriorating mental health among medical students. However, the possible mediating role of resilience in the relationships between personality traits and anxiety has still not yet been explored.

Medical students are of vital importance for the health care service in the future. While China witnessed dramatic enrollment expansion of college students in the past decade, no studies on anxiety symptoms among medical students have yet been carried out. Thus, this study was conducted with the following three aims: 1) to investigate the anxiety status among Chinese medical students; 2) to examine the associations between big five personality traits and anxiety symptoms; 3) to explore the mediating role of resilience in the associations between big five personality traits and anxiety symptoms among Chinese medical students.

## Materials and Methods

### Ethics statement

This study was approved by the Committee on Human Experimentation of China Medical University, the Committee on Human Experimentation of Dalian Medical University, the Committee on Human Experimentation of Shenyang Medical College and the Committee on Human Experimentation of Liaoning Medical College. Written/oral informed consent was obtained according to the Declaration of Helsinki (59th WMA General Assembly, 2009). The consent procedures were approved by all the four Committees on Human Experimentation at the four medical colleges and universities before the survey was conducted.

### Study design and sample

This cross-sectional study was performed in Liaoning province (with a population of about 44 million), China in June 2014. All of the four medical tertiary institutions in the province were included in the investigation, which were Shenyang Medical College, Liaoning Medical College, Dalian Medical University and China Medical University respectively. Medical education in China mainly consists of 5-year programs, 7-year programs and postgraduate education. The students in 5-year programs are awarded bachelor degrees in medicine upon graduation, while those in 7-year programs are awarded master degrees in medicine upon graduation. As more medical students enrolled in 5-year programs, 4 whole classes of clinical medicine students in 5-year programs and 3 whole classes of clinical medicine students in 7-year programs were randomly chosen from each institution based on each academic year. The number of students in each class in Chinese medical schools usually ranges from 25 to 40 in both programs. Self-reported questionnaires were either distributed to the students in the last 20 minutes in class time or sent to the students in later stages of their studies. A total number of 3500 questionnaires were distributed and 3095 (88.43%) students returned the questionnaires in class or to the site coordinators before the deadline. 170 invalid questionnaires were excluded and finally a pool of 2925 students (effective response rate:83.57%) became the subjects.

### Measurement of anxiety symptoms

The Zung Self-Rating Anxiety Scale (SAS), developed by Zung in 1971 [28], was used to measure anxiety symptoms among the students. The SAS questionnaire consists of 20 items and each item is scored on a 4-point Likert scale according to the frequency of the symptoms in the previous week, ranging from 1 (none or a little of the time) to 4 (most or all of the time). The score from each item is calculated to obtain an overall score, and higher overall score indicates higher level of anxiety symptoms. The Chinese version of the questionnaire has been widely used and shows adequate reliability [7,29,30]. The index score of the scale is obtained when the raw score is multiplied by 1.25. A total index score of 50 or higher was used as cutoff point for anxiety symptoms among Chinese population [31]. The Cronbach’s alpha of the scale in the present study was 0.883.

### Measurement of big five personality traits

Big five personality traits were measured by Big Five Inventory (BFI), which was developed by John and Srivastava [32]. BFI measures five personality traits, which are extraversion, agreeableness, conscientiousness, neuroticism and openness. The inventory consists of 44 items. Each item is scored on a 5-point Likert scale ranging from 1 (strongly disagree) to 5 (strongly agree). Each subscale contains 8 to 10 items. The Chinese version of the BFI has been used and demonstrates adequate reliability and validity [33,34]. In the present study, the Cronbach’s alpha coefficients of extraversion, agreeableness, conscientiousness, neuroticism and openness were 0.681, 0.715, 0.652, 0.660 and 0.728 respectively.

### Measurement of resilience

We assessed resilience status using the 14-item Wagnild and Young Resilience Scale (RS-14), which is one of the most reliable tools to measure resilience [35]. It is a 7-point Likert scale used in various age groups and different conditions [36–38]. Each item is graded from 1 (strongly disagree) to 7 (strongly agree). Graded items are summed up to provide a total score and lower scores indicate less resilience. The Cronbach’s alpha of the scale was 0.942 in this study.

### Demographic characteristics

Demographic information regarding age, gender, place of residence, study programs, academic year, and educational levels of both parents were obtained in this study. Place of residence was dichotomized into rural and urban areas. Study programs were divided into 5-year program and 7-year programs. Educational levels of parents were categorized into three groups of primary school, secondary school, college and above.

### Statistical analysis

All analyses were performed using SPSS statistical software for Windows version 13.0 (SPSS, Inc., Chicago, IL). All statistical tests were two-sided and the significance level was set at p<0.05. Descriptive statistics of demographic, personality traits and psychological variables were indicated with mean, standard deviation (SD), number (N) and percentage (%) as appropriate. Pearson’s correlation was used to examine correlations among anxiety, big five personality traits and resilience.

Hierarchical regression analysis was used to explore the effects of groups of independent variables on anxiety symptoms. In step 1, all demographic variables were entered. Because educational levels of parents were categorical variables without a linear trend, dummy variables were set for the two variables respectively. For both paternal and maternal education, “primary school” was set as reference group; in step 2, the five personality traits were entered; in step 3, resilience was added. Standardized estimate (β), F, R² and R²-changes (ΔR²) for each step were provided. Asymptotic and resampling strategies, developed by Preacher and Hayes [39], were used to examine the mediating role of resilience (a*b product) on the associations between personality traits and anxiety symptoms. In these regression equations, personality traits were modeled as predictors, anxiety symptoms as dependent variable, resilience as mediator, and age, gender, place of residence, educational levels of both parents, and study programs as covariates. The bootstrap estimate was based on 5000 bootstrap samples. The bias-corrected and accelerated 95% confidence interval (BCa 95% CI) for each a*b product was calculated, and a BCa 95% CI excluding 0 indicated a significant mediating role. All the continuous variables were standardized in order to avoid multicollinearity [40] before the regression analyses were performed.

## Results

### Demographic characteristics of studied population and prevalence of anxiety

The demographic characteristics of the medical students and the distributions of anxiety symptoms in categorical variables are shown in Table 1. Among the 2925 students, 1028 (35.15%) were males, while 1897 (64.85%) were females. Their age ranged from 15 to 28 (M = 21.65, SD = 1.95). The prevalence of anxiety symptoms among Chinese medical students in the present study was 47.3% (SAS index score≥50).

**Table 1:** Demographic characteristics and differences in anxiety symptoms (N = 2925).

Variables	N	%	SAS (Mean ±SD)
Gender			
Male	1028	35.15%	51.67±12.69
Female	1897	64.85%	48.09±12.02
Age			
15–21	1406	48.07%	47.60±12.28
22–28	1519	51.93%	50.97±12.24
Place of Residence			
Urban area	1809	61.85%	49.63±12.45
Non-urban area	1116	38.15%	48.89± 12.25
Paternal education			
Primary school	301	10.29%	50.28±12.13
Middle School	1450	49.58%	48.70±12.27
College and above	1174	40.13%	49.92±12.53
Maternal Education			
Primary school	469	16.04%	50.32± 12.27
Middle School	1524	52.10%	48.74± 12.20
College and above	932	31.86%	49.86±12.67
Study programs			
Five years	1738	59.42%	51.10 ±12.09
Seven years	1187	40.58%	46.80± 12.34

SAS: the Zung Self-Rating Anxiety Scale

### Correlations among anxiety, big five personality traits and resilience

The correlations among anxiety, big five personality traits and resilience are shown in Table 2. As revealed in the table, the four traits of extraversion, agreeableness, conscientiousness and openness were all negatively related to anxiety symptoms whereas neuroticism was positively related to anxiety symptoms. The results in the table also demonstrate that extraversion, agreeableness, conscientiousness and openness were all positively related to resilience while neuroticism was negatively related to it.

**Table 2:** Means, standard deviation (SD) and correlations of continuous variables.

Variables	Mean	SD	1	2	3	4	5	6	7
1. Anxiety	49.35	12.37	1						
2. Extraversion	24.62	4.86	−0.097**	1					
3. Agreeableness	32.22	5.23	−0.487**	0.138**	1				
4.Conscientiousness	28.84	4.87	−0.231**	0.226**	0.319**	1			
5. Neuroticism	23.54	4.73	0.294**	−0.292**	−0.382**	−0.349**	1		
6. Openness	32.83	5.73	−0.204**	0.349**	0.270**	0.287**	−0.151**	1	
7. Resilience	69.33	15.66	−0.451**	0.227**	0.456**	0.419**	−0.268**	0.361**	1

**P < 0.01.

### Associations between big five personality traits, resilience and anxiety

The results of the hierarchical regression of anxiety symptoms are presented in Table 3. Each step of the independent variables made a significant contribution to the variance in anxiety symptoms. While the demographic factors of age, gender, place of residence, educational levels of parents and study programs contributed to 7.9% of the variance in anxiety symptoms, big five personality traits accounted for 21.3% of the variance in the symptoms. After controlling for the demographic characteristics, four dimensions were significantly related to anxiety symptoms with the exception of extraversion (P > 0.05). Specifically, agreeableness (β = −0.363, P < 0.01), openness (β = −0.066, P < 0.01), and conscientiousness (β = −0.052, P < 0.01) were all negatively associated with anxiety symptoms whereas neuroticism (β = 0.135, P < 0.01) was positively associated with the symptoms.

**Table 3:** The results of hierarchical linear regression analyses.

Variables	Step 1 (β)	Step2 (β)	Step3(β)
**Step 1**			
Age	0.159**	0.103**	0.093**
Gender	−0.138**	−0.103**	−0.087**
Place of residence	−0.026	−0.008	−0.004
Father education 1	−0.053	−0.048	−0.048
Father education 2	−0.008	−0.018	−0.029
Mother education 1	−0.038	−0.020	−0.030
Mother education 2	−0.036	−0.024	−0.028
Study programs	−0.200**	−0.134**	−0.110**
**Step 2**			
Extraversion		0.032	0.048**
Agreeableness		−0.363**	−0.288**
Conscientiousness		−0.052**	0.015
Neuroticism		0.135**	0.128**
Openness		−0.066**	−0.016
**Step 3**			
Resilience			−0.266**
F	31.343**	92.566**	106.627**
R^2^	0.079	0.292	0.339
ΔR^2^	0.079	0.213	0.047

**P < 0.01.

Father education 1 = middle school/primary school; Father education 2 = college and above/primary school; Mother education 1 = middle school/primary school; Mother education 2 = college and above/primary school.

The effect of resilience on anxiety symptoms was significantly negative (β = −0.266, P < 0.01), explaining an additional 4.7% of the variance.

### The mediating role of resilience in the relationships between big five personality traits and anxiety

Path coefficients, size effects of mediator (a*b products), and BCa 95% CI of these products are presented in Table 4. The dimension of extraversion was not significantly related to anxiety symptoms (c path), which was consistent with the result in step 2 of the hierarchical regression. However, after resilience was added as a mediator, extraversion was significantly correlated with anxiety symptoms (c’ path). Thus, there seemed to be a suppressor effect in operation. Because neuroticism was not significantly associated with resilience (a path), resilience did not mediate the association between neuroticism and anxiety symptoms in our study, even though resilience was significantly and negatively associated with anxiety symptoms (b path) after controlling for the predictor variables. Thus, resilience significantly mediated the associations of agreeableness (a*b = −0.075, BCa 95% CI: −0.090, −0.062, p < 0.01), conscientiousness (a*b = −0.066, BCa 95% CI: −0.080, −0.054, p < 0.01) and openness (a*b = −0.050, BCa 95% CI: −0.061, −0.040, p < 0.01) with anxiety symptoms.

**Table 4:** Mediating role of resilience on the associations between personality traits and anxiety symptoms.

Predictors	Path coefficients				a*b (BCa 95% CI)
	c	a	B	c’	
Extraversion	0.032	0.060**	−0.266**	0.049**	−0.016** (−0.025, −0.007)
Agreeableness	−0.363**	0.282**	−0.266**	−0.288**	−0.075** (−0.090, −0.062)
Neuroticism	0.135**	−0.027	−0.266**	0.128**	0.007** (−0.002, 0.017)
Openness	−0.066**	0.188**	−0.266**	−0.016	−0.050** (−0.061, −0.040)
Conscientiousness	−0.052**	0.249**	−0.266**	0.015	−0.066** (−0.080, −0.054)

** p<0.01.

c: associations of personality traits with anxiety symptoms;

a: associations of personality traits with resilience;

b: associations of resilience with anxiety symptoms after controlling for the predictor variables;

c’: associations of personality traits with anxiety symptoms after adding resilience as mediator.

## Discussion

This is one of the few studies to investigate anxiety status among Chinese medical students, to examine the correlations between big five personality traits and anxiety among medical students, and it is the first study to explore the mediating role of resilience in the relationships. This study showed that the prevalence of anxiety symptoms among Chinese medical students was 47.3%, which was higher than that of health care professionals whose anxiety was measured by the same scale. Previous studies demonstrated that the prevalence of anxiety symptoms among doctors and nurses in China was 25.67% [29] and 43.4% [7] respectively. It is worth noting that the prevalence of anxiety symptoms among Chinese medical students was significantly higher than that (12.5%) of nearly two decades ago [41]. Meanwhile, some recent studies revealed that the prevalence of anxiety among medical students was 29.4% in Israel [4], 43.7% in Pakistan [3], 44% in Malaysia [5] 56% in India [6]. The high prevalence of anxiety symptoms among Chinese medical students might be due to the tough environments inherited in medical education, such as academic stress [42]. In addition, in recent years, China has witnessed increasing numbers of reported serious physician-patient conflicts [43], which might exert negative effects on the concerns about their future career among the medical students.

After adjustment for the demographic characteristics, four dimensions of big five personality traits except extraversion were significantly correlated with anxiety symptoms. This is consistent with some literature on the relationship between extraversion and anxiety. In the longitudinal study by Gramstad et al. [18], extraversion of medical students was not significantly related to anxiety when they started to work as junior doctors despite its significant correlation with depression. In contrast, some other studies demonstrated that many anxiety symptoms and disorders, especially social phobia, were related to low extraversion [19]. In step 2 of the regression, based on the absolute value of β, agreeableness, neuroticism, openness and conscientiousness accounted for the variance in anxiety symptoms. It is noteworthy that agreeableness was much more strongly associated with anxiety symptoms than neuroticism. Agreeableness reflects individual differences in people’s interest in needs and well-being of others, and is marked by altruism, social adaptability, likability and emotional support [32]. Agreeable individuals are particularly motivated to avoid emotions which may lead to interpersonal conflicts [44]. These characteristics of agreeableness are negatively related to anxiety symptoms and disorders [45]. More importantly, the strong association between agreeableness and anxiety symptoms may also be related to the traditional Chinese collectivistic culture. The previous research revealed that Taiwanese self-enhanced more for collectivist traits, such as agreeableness, than individualist traits [46]. In such a culture, those who often fail to agree with others can encounter more barriers and obstacles in their life, which may in turn lead to anxiety symptoms. Neuroticism is probably the most studied dimension among the five personality traits and it has been consistently proved to be a significant negative predictor of mental health, such as anxiety and depression in almost all relevant studies [14,16–19,27,47]. Compared with agreeableness and neuroticism, the standardized effect sizes of conscientiousness and openness on anxiety symptoms were much smaller. With regard to conscientiousness, in the meta-analysis by Bogg and Roberts [48], they revealed that conscientiousness was positively related to all beneficial health-related behaviors and negatively related to all risky health-related behaviors and outcomes, including a variety of mental illnesses. Kotov et al. [19] also confirmed the inverse correlation between conscientiousness and mental health, including anxiety and depression. It is shown that individuals scoring high on conscientiousness are better able to automatically down-regulate negative affects, such as anxiety [49]. These people often report a sense of competence and confidence, which may partially account for better mental well-being [50]. One key feature of openness is plasticity, which provides a mechanism whereby responses to internal and external dynamic circumstances can be facilitated [51]. People with high levels of openness may engage more frequently in activities that stimulate and enhance several aspects of their cognitive functioning, which in turn can reduce anxiety [52]. In addition, various cognitive therapies have been proved to effectively reduce levels of anxiety symptoms [53]. Even though personality traits cannot be changed over a short period of time, there is increasing recognition that personality traits are malleable and dynamic constructs, which develop over a lifespan and change in response to maturation and life circumstances rather than a static set of traits [54]. Thus, big five personality traits may function as forecast indicators of anxiety by identifying individuals at risk. The personality traits model could also be used to assess the effects of certain diseases on personality traits. More research is needed to focus on the nature of personality-anxiety relations, which could significantly facilitate the development of preventive interventions.

Big five personality traits were not only directly related to anxiety, but also indirectly related to it through resilience. Resilience was found to mediate the relationships between agreeableness/openness/conscientiousness and anxiety symptoms. Higher scores on agreeableness, conscientiousness and openness among the medical students were associated with higher levels of resilience, which was correlated with lower levels of anxiety symptoms. Meanwhile, higher scores on neuroticism among the students were associated with lower levels of resilience, which correlated with higher levels of anxiety symptoms. The results imply that intervention strategies could focus on not only personality traits, but also the cultivation of resilience. The growing understanding of resilience in different fields has significant implications for the prevention and treatment of anxiety [26]. Based on the theory of social determinants of health, Khanlou and Wray proposed a whole community approach, which indicates that family, school, community and social factors all could make an important contribution to resilience building [55]. Luthara and Brownb pointed out that relationships lied at the “roots” of resilience: the presence of support, love, and security fostered resilience by reinforcing people's innate strengths [56]. Therefore, university authorities could adopt evidence-based measures to enhance resilience of medical students so as to prevent and reduce their anxiety symptoms.

Several limitations of the current study need be mentioned. First, due to the nature of cross-sectional study, causal relations among study variables could not be drawn. The findings from this study should be confirmed by prospective cohort studies in the future. Second, all data was obtained through self-reported questionnaires, which could introduce response bias. The participants might have underestimated or overestimated the relationship among the variables. Since no “gold standard” instrument of measuring resilience is currently available [22], the use of RS-14, which measures personal competence and acceptance of self and life [57], might also have bias. There are some other scales to measure resilience, such as the Connor-Davidson Resilience Scale (CD-RISC) and Psychological Resilience. Five aspects of resilience were measured by CD-RISC, including personal competence, trust/tolerance/strengthening effects of stress, acceptance of change and secure relationships, control and spiritual influences, while three domains of resilience including self-esteem, personal competence and interpersonal control, were measured by Psychological Resilience [22]. Thirdly, we have to acknowledge that the mediating effect of resilience in the relationships between big five personality traits and anxiety symptoms among the subjects in our study was small, which may limit the practical application of our results to some extent. Fourthly, given our study sample, the generalization of the results should be taken with caution. More research should be conducted in other schools and cultures as well.

## Conclusion

This study showed high prevalence of anxiety symptoms among Chinese medical students (47.3%). After adjusting for the demographic factors, agreeableness, conscientiousness and openness were all negatively associated with anxiety symptoms whereas neuroticism was positively associated with anxiety symptoms. Resilience functioned as a mediator in the relationships between agreeableness/conscientiousness/openness and anxiety symptoms. Identifying at-risk individuals and undertaking appropriate intervention strategies that focus on both personality traits and resilience may be more effective in preventing and reducing anxiety symptoms among Chinese medical students.
